# Effect of exogenous spermidine on floral induction, endogenous polyamine and hormone production, and expression of related genes in ‘Fuji’ apple (*Malus domestica Borkh*.)

**DOI:** 10.1038/s41598-019-49280-0

**Published:** 2019-09-04

**Authors:** Ling Qin, Xin Zhang, Jie Yan, Lu Fan, Chunxiao Rong, Chuanyuan Mo, Manrang Zhang

**Affiliations:** College of Horticulture, Northwest A&F University, Yangling, 712100 Shaanxi China

**Keywords:** Shoot apical meristem, Physiology

## Abstract

Flower bud formation in ‘Fuji’ apple (*Malus domestica Borkh*.) is difficult, which severely constrains commercial production. Spermidine (Spd) plays an important role in floral induction, but the mechanism of its action is incompletely understood. To investigate the effect of Spd on flowering, 6-year-old ‘Fuji’ apple trees were treated with 1 × 10^−5^ mol L^−1^ Spd to study the responses of polyamines [putrescine (Put), Spd and spermine (Spm)], hormones [gibberellins (GA_3_) and abscisic acid (ABA)], and polyamine-, hormone- and flowering-related genes. Spd application promoted flowering during floral induction by increasing *MdGA2ox2* (*gibberellin 2-oxidase*) through GA_3_ reduction and increasing *MdNCED1* and *MdNCED3* (*9-cis-epoxycarotenoid dioxygenase*) through ABA enrichment during 60 to 80 days after full bloom. The flowering rate as well as the expressions of flower-related genes, except for *MdLEY* (*LEAFY*), also increased, thereby promoting flowering. In addition, spraying with Spd significantly increased the contents of endogenous polyamines except for Spm in terminal buds by increasing the expressions of polyamine-associated genes. We hypothesize that the contribution of Spd to flowering is related to crosstalk among polyamines, hormone signals, and related gene expressions, which suggests that Spd participates in the apple floral induction process.

## Introduction

Flower bud formation is difficult in ‘Fuji’, the main apple variety in China, which severely hinders commercial production. Floral induction, the most pivotal stage in flower development, is regulated by environmental conditions as well as internal factors such as endogenous hormones^1^. Floral induction ensures that flowering occurs at the proper time in response to complex network processes and multiple environmental signals^2^. Exploration of the regulatory mechanism underlying floral induction is thus crucial to maintain high yield. *SOC1(SUPPRESSOR OF OVER- EXPRESSION OF CONSTANS1)*, *FT(FLOWEING LOCUS T)*, and *LFY(LEAFY)*, which were first discovered in *Arabidopsis thaliana*, are the three core genes in the floral induction pathway^3–5^. *FD(FLOWERING LOCUS)*, like *FT* and *SOC1*, is another key flowering gene. Studies have shown that *SOC1* modulates photoperiod, ambient temperature, and vernalization pathway signals^6,7^. *SOC1* has a central role in the regulation of flowering time and is widely expressed in roots, leaves, and shoot apexes^7,8^. As a characteristic floral meristem gene, *LFY* functions in the floral development regulatory network^9,10^.

Polyamines (PAs), which are aliphatic, low molecular weight, polycationic compounds, act as nitrogenous growth regulators and are considered to be ubiquitous in all living organisms^11^. The major forms of free PAs are putrescine (Put), spermidine (Spd), and spermine (Spm). In plants, these PAs play a crucial role in biological processes throughout the life cycle^12,13^. Because they are positively charged, PAs can interact with cell macromolecules, such as DNA, RNA, chromatin, and phospholipids, as well as with proteins; this interaction explains their relationship to multiple basic cellular processes, including gene expression regulation, translation, chromatin remodeling, RNA processing, protein activation, cell proliferation, cell signaling regulation, membrane stabilization, and modulation of cell death^12,14,15^. Furthermore, numerous studies have shown that PAs are indispensable in advanced plant growth and development processes, including floral bud differentiation, flowering regulation, reproductive organ development, sex differentiation, and fruit growth and senescence^16–18^. Five key biosynthetic enzymes involved in PA biosynthesis in higher plants have currently been investigated: arginine decarboxylase (ADC), ornithine decarboxylase (ODC), S-adenosylmethionine decarboxylase (SAMDC), Spd synthase (SPDS), and Spm synthase (SPMS)^19^. The significant physiological functions of endogenous PAs in floral induction and flower primordial and organ formation have been confirmed in numerous laboratory studies of horticultural crops^20,21^. The triamine Spd, the most important of the three above-mentioned PAs, is involved in many physiological processes. In *Polianthes tuberosa*^20^, Spd and Cad are markers of flower bud differentiation. Experiments on *Cucurbita pepo* L.^22^ and *Ginkgo biloba*^23^ have also found that Spd predominates during flower bud differentiation. When Spd reaches a certain level, it can promote the initiation of a flowering gene, thereby triggering the synthesis of a special protein and finally the formation of the flower primordium^24^. As a consequence, exogeneous Spd was used in the present study.

Gibberellins (GAs) and abscisic acid (ABA) are significant hormones in plant flowering pathways. In Arabidopsis, GAs play a pivotal role in floral induction, which involves a flowering regulatory pathway independent of GA^25^. Studies have shown that GA has different roles in flowering regulation in different plants. In Arabidopsis, for example, GA accelerates the transition from vegetative development to the first inflorescence stage of reproductive development^26^; in contrast, GA has a negative effect on flower bud formation in woody trees such as mango^27^ and apple^28^. Hedden and Thomas^29^ have reported that the terminal steps of GA biosynthesis are mainly catalyzed by gibberellin 20-oxidase (GA20ox) and gibberellin 3-oxidase (GA3ox), whereas GA catabolism is facilitated by gibberellin 2-oxidase (GA2ox). ABA controls many aspects of development, such as seed dormancy, root and bud structure, flowering time, and senescence^30–33^. The active effect of ABA on flowering time acceleration and drought-escape response has recently been demonstrated in Arabidopsis and rice^32,34^. In transgenic Arabidopsis, *NCED* is reportedly adjusted by positive feedback through ABA^35^. ABFs are a family of ABA-responsive element binding factors. In the presence of ABA, SnRK2 can phosphorylate its downstream target ABFs (i.e., ABF1, ABF2, ABF3, and ABF4)^36,37^.

Given the above relationship of PAs and hormones to floral induction, we studied the molecular mechanism underlying the physiological response of ‘Fuji’ apple to exogenous Spd treatment. To advance understanding of PA and hormonal regulation of flowering, we examined the responses of endogenous PAs and hormones, floral genes, and genes related to PA and hormone synthesis following spraying with Spd before the cessation of shoot growth. We then used the results to propose how Spd regulates floral development in conjunction with floral and related genes. The ultimate goals of this research were to elucidate the effect of PAs on flower formation from a physiological–molecular perspective, further enrich basic biological knowledge of fruit tree flowering, and provide a theoretical basis for artificial regulation of apple flowering time.

## Results

### Effects of exogenous Spd on flowering rate

**Figure 1:**
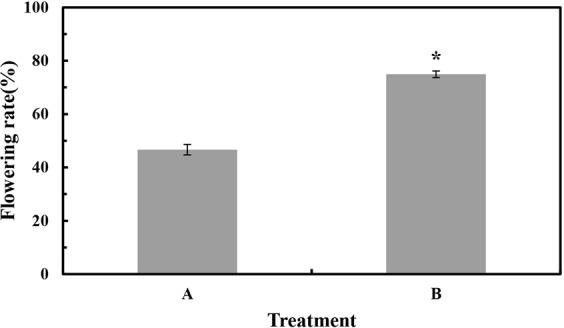
Effect of spermidine (Spd) treatment on the flowering rate (%) of ‘Fuji’ apple. (**A**,**B**) Flowering rates in control (**A**) and Spd-treated trees (**B**). Values are means ± SE (*n* = 3); an asterisk indicates a significant difference (*P* < 0.05) between the two groups.

To study the effect of exogenous Spd on apple flowering, the flowering rate of short shoots was investigated in the second year after treatment. As shown in Fig. 1, the flowering percentage was higher in trees treated with Spd than in the control.

### Effects of exogenous Spd on PA contents

**Figure 2:**
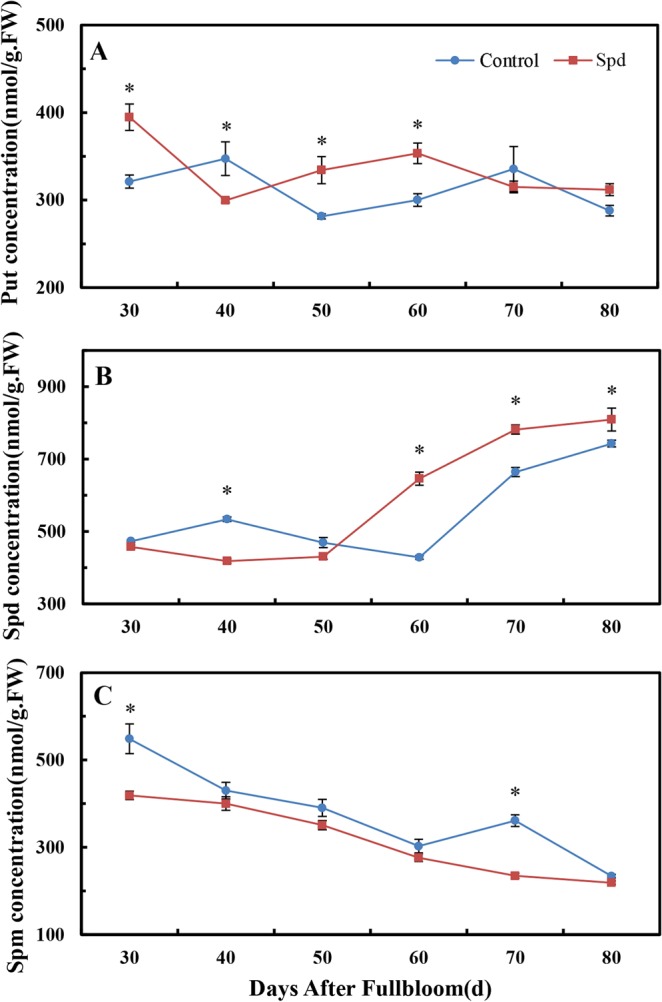
Endogenous polyamine contents of Spd-treated terminal buds of ‘Fuji’ apple. (**A**) Put; (**B**) Spd; (**C**) Spm. Values are means ± SE (*n* = 3); an asterisk indicates a significant difference (*P* < 0.05) between the treatment and control groups.

After applying exogenous Spd, we simultaneously analyzed the contents of the three endogenous PAs, namely, Put, Spd, and Spm. Except at 40 DAF, the Put content of buds treated with Spd was significantly higher than that of the control until 60 DAF, with no significant differences observed thereafter (Fig. 2A). At the early floral induction stage (40 DAF), Spd levels were significantly decreased in treated buds relative to the control and then significantly steadily increased (Fig. 2B). Spm levels, which declined over time in both groups, were significantly lower in the Spd-treated group compared with the control at 30 and 70 DAF (Fig. 2C).

As demonstrated by the results shown in Fig. 1, Spd application significantly affected the contents of endogenous PAs in all tested trees during at least one time point.

### Effects of exogenous Spd on GA3 and ABA contents

**Figure 3:**
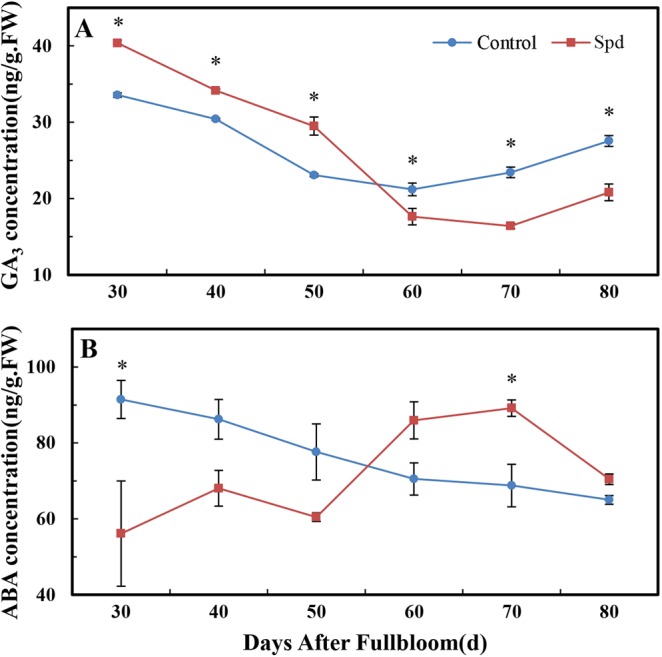
Levels of endogenous hormones in Spd-treated terminal buds of ‘Fuji’ apple. (**A**) GA3; (**B**) ABA. Values are means ± SE (n = 3); an asterisk indicates a significant difference (P < 0.05) between the treatment and control groups.

We studied the effect of Spd treatment on endogenous GA_3_ and ABA concentrations during flower development. Compared with the control, Spd spraying significantly elevated the level of GA_3_ at 30–50 DAF, while GA_3_ content was significantly lower than that of the control group at 60–80 DAF (Fig. 3A). Terminal buds of trees treated with exogenous Spd contained significantly less endogenous ABA at 30 DAF and significantly more at 70 DAF relative to the control (Fig. 3B).

According to the above results, as shown in Fig. 3, the influence of Spd on GA_3_ and ABA contents differed depending on the physiological processes that were active.

### Effects of exogenous Spd on flowering-associated gene expressions

**Figure 4:**
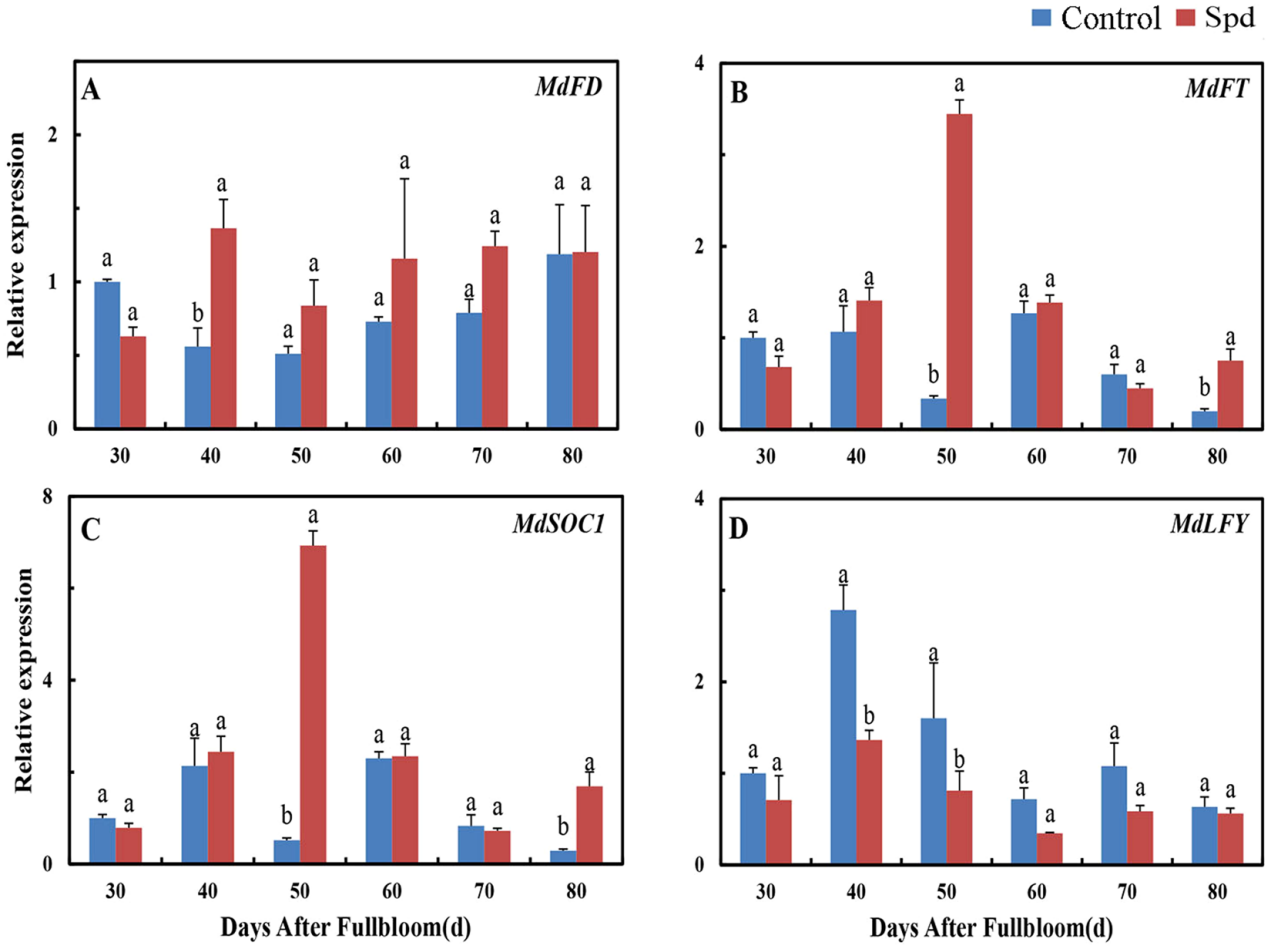
Relative expression levels of floral genes in ‘Fuji’ apple terminal buds during floral induction after Spd treatment. Values are means ± SE (*n* = 3). Means labeled by different lowercase letters (a and b) are significantly different (*P* < 0.05).

To better understand molecular events associated with significant changes in the degree of flowering, we analyzed the expressions of genes controlling flowering in terminal spur buds. *MdFD* transcription was significantly up-regulated at 40 DAF after treatment with Spd (Fig. 4A). The expression pattern of the floral integrator gene *MdFT* was very similar to that of *MdSOC1*, with significant up-regulation and peak expression of both genes observed at 50 DAF in the Spd-treated group (Fig. 4B,C). In contrast, the expression of *MdLFY* remained lower than in the control, with exogenous Spd significantly down-regulating *MdLFY* transcription at 40 and 50 DAF compared with the control (Fig. 4D).

These results (Fig. 4) indicate that exogenous Spd application significantly affected all flowering-related genes at one or more time points, thus suggesting that these genes responded to Spd levels in buds.

### Effects of exogenous Spd on PA-related gene expressions

**Figure 5:**
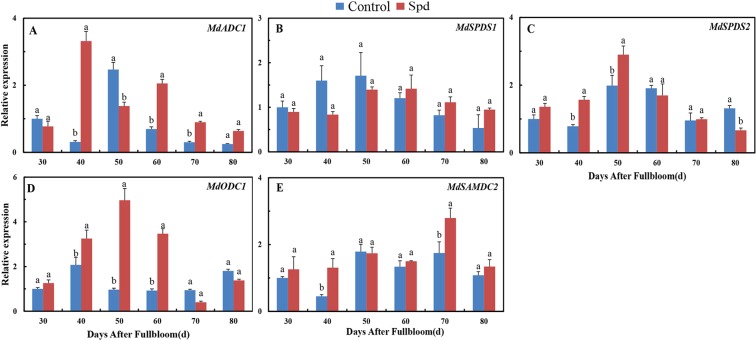
Expression profiles of PA-related genes in ‘Fuji’ apple terminal buds during floral induction after Spd treatment. Values are means ± SE (*n* = 3). Means labeled by different lowercase letters (a and b) are significantly different (*P* < 0.05).

As shown in Fig. 5, transcription levels of PA biosynthetic genes, including *MdADC1*, *MdODC1*, *MdSAMDC2*, *MdSPDS1*, and *MdSPDS2*, changed rapidly after exogenous Spd application. *MdADC1* expression was obviously up-regulated in Spd-treated buds at some time points. In particular, *MdADC1* was up-regulated at 40, 60, and 70 DAF and significantly down-regulated at 50 DAF in shoots treated with Spd compared with the untreated control (Fig. 5A). Interestingly, *MdSPDS1* and *MdSPDS2*, which both belong to the *MdSPDS* gene family, had different responses to Spd treatment. Exogenous Spd decreased transcript levels of *MdSPDS1* and significantly increased those of *MdSPDS2* at 40 and 50 DAF, whereas application of this PA respectively increased and decreased transcript levels of *MdSPDS1* and *MdSPDS2* from 60 to 80 DAF (Fig. 5B,C). A significant increase in *MdODC1* transcript levels was observed in Spd-treated shoots from 40 to 60 DAF (Fig. 5D). In addition, the expression of *MdSAMDC2* in Spd-treated buds mainly increased during bud development and peaked at 70 DAF (Fig. 5E).

### Effects of exogenous Spd on expressions of GA- and ABA-related genes

**Figure 6:**
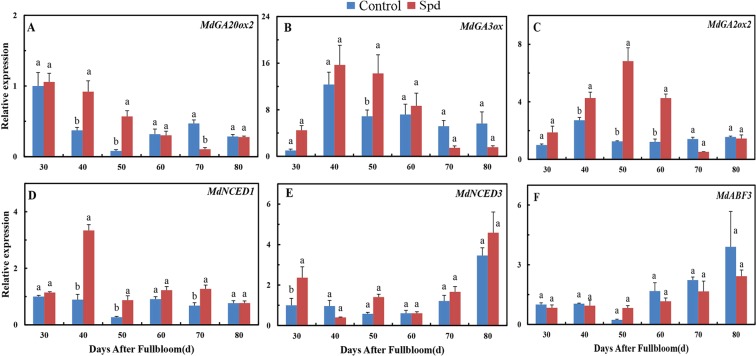
Expression patterns of GA- and ABA-related genes in ‘Fuji’ apple terminal buds during floral induction after Spd treatment. (**A**–**F**) Expression patterns of genes related to GA (**A**–**C**) and ABA (**D**–**F**). Values are means ± SE (*n* = 3). Means labeled by different lowercase letters (a and b) are significantly different (*P* < 0.05).

We analyzed two important synthetic genes in the gibberellin biosynthesis pathway. *MdGA20ox2* and *MdGA3 oxidase* (*GA3ox*) expressions in Spd-treated buds were initially significantly higher than in control buds, with *MdGA20ox2* and *MdGA3ox* subsequently down-regulated at 70 and 80 DAF (Fig. 6A,B). In addition, the expression level of *MdGA2ox2*, a gene involved in the inactivation of GA catabolites, was also high. In particular, *MdGA2ox2* exhibited significantly up-regulated expression from 40 to 60 DAF in response to Spd treatment (Fig. 6C). These data indicate the possible existence of a negative feedback loop maintaining a GA steady state^29^.

Several ABA signaling pathway genes were also analyzed, including *NCED*, a family of ABA biosynthetic enzymes^38^. *NCED1* and *NCED3* are key regulatory factors in ABA biosynthesis. In addition, *ABF3* is a major transcription factor in the ABA signaling pathway. Notably, expression levels of *MdNCED1* and *MdNCED3* substantially increased in response to exogenous Spd. *MdNCED1* expression peaked at 40 DAF, and *MdNCED3* levels were highest at 80 DAF (Fig. 6D,E). In contrast, expression of *MdABF3* decreased, but the difference was not significant. (Fig. 6F).

## Discussion

Numerous studies have revealed that flower bud differentiation is affected by a combination of *in vitro* and *in vivo* factors. Evidence suggesting a close connection between PAs and the physiological events leading to flowering has been summarized by Galston and Kaur-Sawhney^39^, Kakkar and Rai^40^, and Galston *et al*.^41^. In the present study, we investigated the roles of PAs in flower development in ‘Fuji’ apple. We observed a correlation between the concentration of endogenous PAs and hormones and the expression of related genes during floral development.

### Effect of exogenous Spd on flower formation

The application of Spd has been previously shown to accelerate flower bud formation^20^, a finding further confirmed by our experimental results (Fig. 1). After spraying with Spd, the flowering rate of short shoots increased, which suggests that Spd treatment is beneficial to flower bud formation. At the molecular level, plant flowering is regulated by complicated networks involving endogenous and environmental signals^42^. In our study, the transcriptional activities of floral integrators *MdFD* (Fig. 6A), *MdFT* (Fig. 6B), and *MdSOC1* (Fig. 6C) were enhanced by exogenous Spd. A rise in transcription levels of an *FT*-encoding gene has been connected with floral induction in several species^43^. According to our results, Spd applied before floral induction can hasten flowering in ‘Fuji’ apple. To our surprise, we observed a reduction in *MdLFY* expression during floral induction in apple buds treated with Spd (Fig. 5D). We speculate that *MdLFY* is subject to other modes of regulation in apple, but this hypothesis requires further testing.

### PA signaling regulates floral induction in Spd-treated buds

The significance of PAs in flowering development has received much attention^44^. Because PA biosynthesis is transcriptionally regulated, we analyzed the expressions of genes related to PA biosynthesis in Spd-treated shoots. As inferred from results displayed in Figs 2A–C and 5A–F, we discovered that exogenous Spd significantly impacted the contents of endogenous PAs and related gene expressions in all tested materials at some time point(s). According to our results, Spd boosted the level of endogenous Spd in buds during the late stage of floral induction (from 60 to 80 DAF); however, no significant difference was found in *MdSPDS1* expression during floral induction, and *MdSPDS2* expression even significantly declined. The increase in endogenous Spd levels in Spd-treated buds may be mainly due to the permeation of exogenous Spd in addition to the synthesis of new Spd. In plants, Put is biosynthesized from arginine via ADC or from ornithine by way of ODC^20^. We propose that the presence of Spd augmented the content of Put because exogenous Spd significantly increased *MdADC1* and *MdODC1* expression and even *MdSAMDC2* transcripts. Conversely, we observed a depression in the Spm content of Spd-treated buds, a finding in accord with previous studies where exogenous Spd decreased endogenous PA contents during flowering^45^. We conclude that feedback inhibition caused by high levels of endogenous PAs takes place after application of exogenous Spd. This finding contrasts with the results of Huang^20^, who found that early floral initiation is associated with a rise in free Spd and a drop in free Put and Spm in *P. tuberosa*. These conflicting results suggest that the effects of PAs on flowering vary depending on species, organ, and physiological process. Our results collectively demonstrate that endogenous PA contents and the expressions of related genes change markedly and regularly after exogenous Spd application along with developmental stages of flower bud differentiation, thereby affecting flowering.

### Expression of GA genes in Spd-treated buds

To provide evidence for the influence of applied Spd on the metabolism of endogenous hormones in terminal spur buds, we measured endogenous hormone concentrations and transcript levels of hormone biosynthetic genes. Hormones synergistically or antagonistically play a pivotal regulatory role in plant growth and development^1^. GA is one of the most studied hormones in the flowering process in plants, and the mutual effect of PAs and GAs has been preliminarily researched in species such as Arabidopsis and tomato^46–48^. Alcázar *et al*.^46^ have reported that a high concentration of endogenous Spd/Spm in *35S:AtADC2* Arabidopsis plants is accompanied by a significant drop in *AtGA3ox3* and *AtGA20ox1* expression and a decrease in GA production. In addition, a significant increase in *GA2ox* expression and a decrease in GA content have been found in *E8:ySAMDC* transgenic tomato fruits, which had three to four times more Spd compared with the control^48^. In our study, however, we discovered that Spd application dramatically augmented the GA content of buds by increasing transcript levels of the GA biosynthetic genes *MdGA20ox2* and *MdGA3ox* during the initial process of floral induction (30 to 50 DAF) while simultaneously significantly increasing transcript levels of the GA catabolism gene *MdGA3ox*. This result suggests the existence of a negative feedback loop to maintain GA balance^29^. Next, we found that Spd application lowered the expressions of *MdGA20ox2* and *MdGA3ox* and increased the expression of *MdGA2ox2*, which significantly restrained GA biosynthesis from 60 to 80 DAF. Hence, the effects of Spd on GA vary depending on physiological processes, and gene family members have diverse reactions to Spd stimulation. As a high GA content inhibits flowering^49^, we conclude that exogenous Spd promotes flower formation by reducing GA in the later stages of physiological differentiation. The specific mechanism remains unclear, however, and requires further research.

### Expression of ABA genes in Spd-treated buds

A previous finding that ABA contents of leaves and shoots change significantly during the induction phase of apple flowering^50^ suggests that ABA participates in the regulation of flowering induction in perennial woody plants. *NCED3* has different functions in different species. In *Brassica napus*, for example, *BnNCED3* has been discovered to participate in both stress adaptation and plant development^51^. After rapid induction of *MdNCED1* expression in apples, endogenous ABA content gradually increases^52^. The interplay between ABA and PAs during seed maturation and germination has also been thoroughly researched^53^. Further evidence highlighting the interaction between PA anabolism and the ABA signaling pathway has been obtained in grape, a perennial plant^46^. ABA content is positively correlated with the expression level of *NCED*^51^. Our data indicate that Spd application significantly enhances the expressions of *MdNCED1* and *MdNCED3*, in turn increasing ABA levels during the late stage of floral induction. ABF3 has redundant functions in ABA signaling^54^. In our study, exogenous Spd decreased the expression of *MdABF3*. We thus infer that Spd regulates flowering by depressing *MdABF3* expression. Results reported by Keumbi Hwang *et al*.^55^ support the idea that ABA is involved in flowering regulation to speed the floral transition. Increased ABA is thus considered to play a crucial role in flowering regulation. Our results indicate that Spd promotes flowering to a certain extent and is closely related to ABA metabolic regulation at the transcriptional level.

## Conclusions

**Figure 7:**
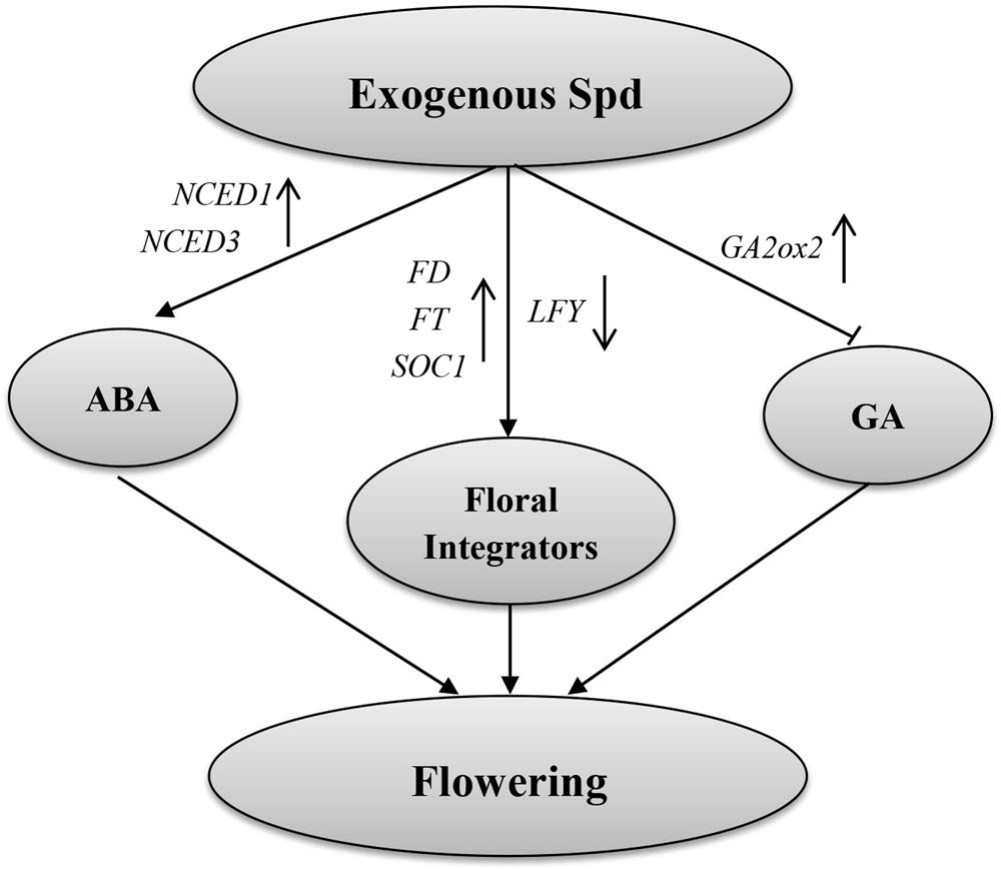
A schematic model to explain our experimental results. Arrows between processes correspond to promotion, while the line with a terminal bar indicates inhibition.

The current study revealed the existence of an intricate regulatory mechanism that manages floral induction in ‘Fuji’ apple. The contribution of Spd to the promotion of flowering might be closely associated with the metabolism of hormones and floral integrators (Fig. 7). How PAs regulate flowering genes and influence ‘Fuji’ flowering, however, still needs further study.

## Materials and Methods

### Plant materials, growth conditions, and Spd treatment

All experiments were carried out at the Apple Experimental Station of Northwest A&F University, Qianyang, Shanxi Province, China (107.13°E, 34.65°N). Six-year-old trees of ‘Fuji’/M26/*M. robusta* Rehd. were chosen at random and divided into six blocks of three trees each, with a spacing of 1.3 m × 4.0 m. Three blocks were sprayed with 1 × 10^−5^ mol L^−1^ Spd (Sigma Chemical Company). The other three blocks were sprayed with water and used as a control. All treatments were carried out with a low-pressure hand-wand sprayer. The spraying was performed twice on clear mornings at 22 and 28 days after full bloom (DAF) (April 27 and May 3, respectively). Terminal buds on spurs (<5 cm) were collected into liquid nitrogen at 30, 40, 50, 60, 70, and 80 DAF, stored at −80 °C, and used for analysis of PAs, hormone quantification, and correlation analysis of gene expression.

### Analysis of flowering rate

The flowering rate was calculated as described by Zuo *et al*.^56^ with sight modifications. Specifically, two large branches on each Spd-treated or control tree were labeled, and the flowering rate of terminal buds on their short shoots (<5 cm) were recorded. During full bloom, on April 10, 2019, the total number of terminal buds on short shoots and the number of floral terminal buds on the marked branches were counted. The flowering rate (number of floral buds/number of total buds) based on these data was then calculated.

### Extraction and measurement of endogenous PAs in spur apical buds

PAs were estimated using high-performance liquid chromatography (HPLC) as described by Flores and Galston^57^ with minor modifications. Briefly, frozen tissue (0.3 g) from each treatment was mixed with 3 mL cold 5% (w/v) perchloric acid in a centrifuge tube. The homogenate was chilled in an ice bath for 1 h and then centrifuged at 15,000 ×*g* for 30 min at 4 °C. Next, 1 mL of 2 M NaOH solution was combined with 1 mL supernatant, and 10 µL benzoyl chloride was added. The mixture was incubated for 20 min at 37 °C, and 2 mL saturated sodium chloride was then added to terminate the reaction. Benzoyl-PAs were extracted into 2 mL diethyl ether by centrifugation at 4,500 ×g for 5 min. 1 mL of the ether phase was collected and evaporated to dryness with a nitrogen blower. The dried extracts were dissolved in 500 µL of 60% methanol for further testing.

The above-prepared benzoyl-PA extract was filtered through a 0.22-μm membrane filter and then eluted on a C18 reverse-phase column (6.0 mm × 150 mm; particle size 5 mm) at room temperature. The mobile phase was composed of HPLC-grade methanol, HPLC-grade acetonitrile, and ddH_2_O (58:2.5:39.5, v/v/v). The flow rate was 1.0 mL/min, the detection wavelength was 254 nm, and the column temperature was 30 °C. Put, Spd and Spm PA standards were used to construct standard curves. The PA analyses were repeated three times.

### Quantitative analysis of endogenous hormones in spur apical buds

GA_3_ and ABA in terminal spur buds were measured by liquid chromatography as described previously^58^ with slight modifications. Specifically, 0.3 g of each treated fresh bud sample in 20 mL of 70% cold acetone was maintained on ice for 1 h. After centrifugation at 8,000 ×g and 4 °C for 10 min, the supernatant was collected and concentrated using a nitrogen evaporator. Next, extraction was performed by addition of 10 mL of petroleum ether, decolorization for 10 min, and removal of the ether phase. This procedure was carried out three times. Ten milliliters of ethyl acetate was added and collected after 10 min, and this step was repeated once more. The two collections of the ester phase were concentrated with a nitrogen blower until no solvent remained. The dried extracts were dissolved in 1.5 mL methanol and filtered through a 0.22-μm filter for future analysis.

The liquid-chromatography mobile phase consisted of HPLC-grade methanol and 0.1% formic acid. The flow rate was 1.0 mL/min, and the column temperature was 30 °C. GA_3_ and ABA (Sigma) standards were used to construct standard curves. Three biological replicates of GA_3_ and ABA analyses were performed.

### RNA isolation and cDNA synthesis from spur apical buds

Total RNA was isolated using a cetyltrimethylammonium bromide method^59^ with minor modifications.

RNA from all samples was quantified spectrophotometrically, and total RNA purity and integrity were assessed by agarose gel electrophoresis. Total RNA (1 µg) was reverse transcribed into cDNA using a PrimeScript RT Reagent kit with gDNA Eraser (Takara, Shiga, Japan) according to the manufacturer’s instructions.

### Gene expression analysis by quantitative RT-PCR (RT-qPCR)

Gene-specific primers were used for PCR amplification (Table 1). RT-qPCR amplifications were performed in 20-μL reaction mixtures containing 2 μL cDNA (diluted 1:16), 10 μL of 2 × SYBR Premix ExTaq II (Takara), 0.8 μL of each primer, and 6.4 μL ddH_2_O. The RT-qPCR assay was carried out on a QuantStudio5 instrument. The cycling protocol was as follows: 30 s of denaturation at 95 °C, followed by 40 cycles of 95 °C for 5 s, 55 °C for 30 s, and 72 °C for 30 s. A melting curve analysis was performed immediately after the PCR amplification.

**Table 1:** Primers used for quantitative real-time PCR gene expression analysis.

Gene accession no.	Primers (forward/reverse)	Sequence (5′-3′)
MDP0000169473	*MdFD-*F	AGTGACCAGACCAACCACAACA
	*MdFD-*R	ATTTGGGTGGTGGGATCAGTGA
MDP0000132050	*MdFT-*F	TGGTGGAGACGATCTCAGGACT
	*MdFT-*R	TTGCCGCAGTAGTTGCTGGAAT
MDP0000144597	*MdSOC1-*F	GTGCGAAGCCGTGGATGGAG
	*MdSOC1*-R	CAACAGCGCGCACCATTTGGC
MDP0000186703/MDP0000861601	*MdLFY*-F	TTGGCGGCAGGCATGTTACA
	*MdLFY-*R	GCTAGAGGCAGTGGCGTTGTT
MDP0000161317	*MdGA3ox-*F	TGAGATGCTGAGCAACGGAGTT
	*MdGA3ox-*R	CGCAACCGACACCCTTTCTTTC
MDP0000248981	*MdGA20ox2-*F	CAGGGCAGTGGTGAACAGTGAA
	*MdGA20ox2-*R	TTGTGAACTCCAGCAGCATCGA
MDP0000139968/MDP0000950387	*MdGA2ox2-*F	TTATGGCTGCAAAGGCGACACT
	*MdGA2ox2-*R	TGCCCATCCATTCTCACTCCCT
MDP0000813805/MDP0000333494	*MdNCED1-*F	CTGAAACAGGGGACCTCAAA
	*MdNCED1-*R	CGTAGCTAAGCGCGAAGAGT
MDP0000228070	*MdNCED3-*F	ACAAGACACCGCCACCTTTC
	*MdNCED3-*R	TTGGGATTTGGATTACAGAAGG
MDP0000248567	*MdABF3-*F	ACATCTTCATTGTCGCCGGT
	*MdABF3-*R	AGGTATAGGCCTGTTTACGAGC
MDP0000813339	*MdADC1-*F	GATAGCTCTCTTCCCGCGTC
	*MdADC1-*R	CGATTCGGTAGAGGTCGGAT
MDP0000228682	*MdODC1-*F	AGCTTGGCGTAATCATGGTC
	*MdODC1-*R	ATGCTTCCGGCTCGTATGTT
MDP0000914975	*MdSAMDC2-*F	CAAGTGTACCGTGGAGCAAT
	*MdSAMDC2-*R	TGCAACCATTGTGAAAGCCA
MDP0000185362	*MdSPDS1-*F	GCCTCACGGTGGAATCAAGA
	*MdSPDS1-*R	GCCATGGGGTTATGTTGGGA
MDP0000198590	*MdSPDS2-*F	CGAGCGCCTTTTTATCCCGT
	*MdSPDS2-*R	GGAGGCAAAAACTAGCTGGC
MDP0000027925	*MdADC1-*F	TGGTCCCTGTCTCAGAGTGT
	*MdADC1-*R	GCCAAACGCACCAAAGGAAT
MDP0000912745	*MdACTIN-*F	TGACCGAATGAGCAAGGAAATTACT
	*MdACTIN-*R	TACTCAGCTTTGGCAATCCACATC

Three replicates were analyzed per sample. The MdACTIN gene was used as an internal control for standardization of relative expression levels of all tested genes. Relative gene expression levels were calculated by the 2^−ΔΔCt^ method^60^.

### Statistical analysis

All data, which were represented as means ± SE of three replicates, were subjected to analysis of variance (ANOVA) at the 5% level in IBM SPSS v19. The significance of differences among means was determined by Duncan’s multiple range test. Plots were generated with Excel 2007.

## Data Availability

The datasets generated during and/or analyzed during the current study are available from the corresponding author on reasonable request.

## References

[1] Guitton B (2012). Genetic control of biennial bearing in apple. Journal of Experimental Botany..

[2] Kurokura T, Mimida N, Battey NH, Hytonen T (2013). The regulation of seasonal flowering in the Rosaceae. Journal Of Experimental Botany..

[3] Lee J, Lee I (2010). Regulation and function of *SOC1*, a flowering pathway integrator. Journal Of Experimental Botany..

[4] Fan Chengming, Hu Ruibo, Zhang Xiaomei, Wang Xu, Zhang Wenjing, Zhang Qingzhe, Ma Jinhua, Fu Yong-Fu (2014). Conserved CO-FT regulons contribute to the photoperiod flowering control in soybean. BMC Plant Biology.

[5] Wang JW (2014). Regulation of flowering time by the miR156-mediated age pathway. Journal Of Experimental Botany..

[6] Hepworth SR, Valverde F, Ravenscroft D, Mouradov A, Coupland G (2002). Antagonistic regulation of flowering-time gene SOC1 by CONSTANS and FLC via separate promoter motifs. Embo Journal..

[7] Immink RGH (2012). Characterization of SOC1’s Central Role in Flowering by the Identification of Its Upstream and Downstream Regulators. Plant Physiology..

[8] Hou, X. *et al*. Nuclear factor Y-mediated H3K27me3 demethylation of the SOC1 locus orchestrates flowering responses of Arabidopsis. *Nature Communications*. **5** (2014). 10.1038/ncomms5601 25105952

[9] Blazquez MA, Weigel D (2000). Integration of floral inductive signals in Arabidopsis. Nature..

[10] Blazquez MA, Ahn JH, Weigel D (2003). A thermosensory pathway controlling flowering time in Arabidopsis thaliana. Nature Genetics..

[11] Choubey A, Rajam MV (2017). Transcriptome response and developmental implications of RNAi-mediated ODC knockdown in tobacco. Functional & Integrative Genomics..

[12] Tiburcio AF, Altabella T, Bitrian M, Alcazar R (2014). The roles of polyamines during the lifespan of plants: from development to stress. Planta..

[13] Guo J (2018). Polyamines Regulate Strawberry Fruit Ripening by Abscisic Acid, Auxin, and Ethylene. Plant Physiology..

[14] Kusano T, Berberich T, Tateda C, Takahashi Y (2008). Polyamines: essential factors for growth and survival. Planta..

[15] Baron K, Stasolla C (2008). The role of polyamines during *in vivo* and *in vitro* development. In Vitro Cellular & Developmental Biology-Plant..

[16] Ahmed S (2017). Altered expression of polyamine transporters reveals a role for spermidine in the timing of flowering and other developmental response pathways. Plant Science..

[17] Sorkheh K (2011). Response of *in vitro* pollen germination and pollen tube growth of almond (Prunus dulcis Mill.) to temperature, polyamines and polyamine synthesis inhibitor. Biochemical Systematics And Ecology..

[18] Nambeesan S (2010). Overexpression of yeast spermidine synthase impacts ripening, senescence and decay symptoms in tomato. Plant Journal..

[19] Bagni N, Malucelli B, Torrigiani P (2010). Polyamines, storage substances and abscisic acid-like inhibitors during dormancy and very early activation of Helianthus tuberosus tuber tissues. Physiologia Plantarum..

[20] Huang CK (2004). Changes in polyamine pattern are involved in floral initiation and development in Polianthes tuberosa. Journal Of Plant Physiology..

[21] Guo JE, Li T, Sun X, Zheng C, Sun X (2015). Relationship between Endogenous Polyamines and Floral Bud Differentiation in Chrysanthemum morifolium under Short-day Conditions. Korean Journal Of Horticultural Science & Technology..

[22] Huang Z, Shen H, Xie Y (2002). The changes of endogenous hormones and polyamines of the cotyledons of Cucurbita pepo L. *in vitro* during floral bud differentiation. Nanjing Shida Xuebao (Ziran Kexue Ban)..

[23] Zhang W, Jun HE, Shi J (2002). Changes of endogenous polyamines during differentiation on flower buds of Ginkgo biloba. Journal of Zhejiang Forestry College..

[24] Chen Y, Shen H (1999). Correlation Between Endogenious Polyamine Contents and Flowr Bud Formation in Dianthus Chinensis L. Acta Horticulturae Sinica..

[25] Mutasa-Goettgens E, Hedden P (2009). Gibberellin as a factor in floral regulatory networks. Journal Of Experimental Botany..

[26] Yamaguchi N (2014). Gibberellin Acts Positively Then Negatively to Control Onset of Flower Formation in Arabidopsis. Science..

[27] Nakagawa M, Honsho C, Kanzaki S, Shimizu K, Utsunomiya N (2012). Isolation and expression analysis of FLOWERING LOCUS T-like and gibberellin metabolism genes in biennial-bearing mango trees. Scientia Horticulturae..

[28] Wilkie JD, Sedgley M, Olesen T (2008). Regulation of floral initiation in horticultural trees. Journal Of Experimental Botany.

[29] Hedden P, Thomas SG (2012). Gibberellin biosynthesis and its regulation. Biochemical Journal..

[30] Yao C, Finlayson SA (2015). Abscisic Acid Is a General Negative Regulator of Arabidopsis Axillary Bud Growth. Plant Physiology..

[31] Ondzighi-Assoume CA, Chakraborty S, Harris JM (2016). Environmental Nitrate Stimulates Abscisic Acid Accumulation in Arabidopsis Root Tips by Releasing It from Inactive Stores. Plant Cell..

[32] Riboni M, Test AR, Galbiati M, Tonelli C, Conti L (2016). ABA-dependent control of GIGANTEA signalling enables drought escape via up-regulation of FLOWERING LOCUS T in Arabidopsis thaliana. Journal Of Experimental Botany..

[33] Song, Y. *et al*. Abscisic Acid as an Internal Integrator of Multiple Physiological Processes Modulates Leaf Senescence Onset in Arabidopsis thaliana. *Frontiers In Plant Science*. **7** (2016). 10.3389/fpls.2016.00181 PMC4759271 26925086

[34] Du H (2018). Integrative Regulation of Drought Escape through ABA-Dependent and -Independent Pathways in Rice. Molecular Plant..

[35] Nonogaki M, Sall K, Nambara E, Nonogaki H (2014). Amplification of ABA biosynthesis and signaling through a positive feedback mechanism in seeds. Plant Journal.

[36] Furihata T (2006). Abscisic acid-dependent multisite phosphorylation regulates the activity of a transcription activator AREB1. Proceedings Of the National Academy Of Sciences Of the United States Of America..

[37] Yoshida T (2015). Four Arabidopsis AREB/ABF transcription factors function predominantly in gene expression downstream of SnRK2 kinases in abscisic acid signalling in response to osmotic stress. Plant Cell And Environment..

[38] Cadman CSC, Toorop PE, Hilhorst HWM, Finch-Savage WE (2006). Gene expression profiles of Arabidopsis Cvi seeds during dormancy cycling indicate a common underlying dormancy control mechanism. Plant Journal..

[39] Galston AW, Sawhney RK (1990). Polyamines in plant physiology. Plant physiology..

[40] Kakkar RK, Rai VK (1993). Plant Polyamines in Flowering and Fruit Ripening. Phytochemistry..

[41] Galston AW, KaurSawhney R, Altabella T, Tiburcio AF (1997). Plant polyamines in reproductive activity and response to abiotic stress. Botanica Acta..

[42] Shalom L (2014). Fruit load induces changes in global gene expression and in abscisic acid (ABA) and indole acetic acid (IAA) homeostasis in citrus buds. Journal Of Experimental Botany..

[43] Mimida N (2011). Expression patterns of several floral genes during flower initiation in the apical buds of apple (Malus x domestica Borkh.) revealed by *in situ* hybridization. Plant Cell Reports..

[44] Applewhite PB, Kaur-Sawhney R, Galston AW (2000). A role for spermidine in the bolting and flowering of Arabidopsis. Physiologia Plantarum..

[45] Xu J, Chen H, Ma B, Zhang A (2001). Effects of Exogenous Spermidine on the Levels of Endogenous Hormones and Polyamines in the Flowers and Fruitlets of Red Fuji Apple. Acta Horticulturae Sinica..

[46] Alcazar R, Garcia-Martinez JL, Cuevas JC, Tiburcio AF, Altabella T (2005). Overexpression of ADC2 in Arabidopsis induces dwarfism and late-flowering through GA deficiency. Plant Journal..

[47] Bailly C, El-Maarouf-Bouteau H, Corbineau F (2008). From intracellular signaling networks to cell death: the dual role of reactive oxygen species in seed physiology. Comptes Rendus Biologies..

[48] Kolotilin I (2011). Expressing yeast SAMdc gene confers broad changes in gene expression and alters fatty acid composition in tomato fruit. Physiologia Plantarum..

[49] Zhang S (2016). Effect of exogenous GA(3) and its inhibitor paclobutrazol on floral formation, endogenous hormones, and flowering-associated genes in ‘Fuji’ apple (Malus domestica Borkh.). Plant Physiology And Biochemistry..

[50] Xing L (2015). Transcription Profiles Reveal Sugar and Hormone Signaling Pathways Mediating Flower Induction in Apple (Malus domestica Borkh.). Plant and Cell Physiology..

[51] Xu P, Cai W (2017). Functional characterization of the BnNCED3 gene in Brassica napus. Plant Science..

[52] Kondo S (2012). Dehydration tolerance in apple seedlings is affected by an inhibitor of ABA 8′-hydroxylase CYP707A. Journal Of Plant Physiology..

[53] Antolin MC, Santesteban H, Maria ES, Aguirreolea J, Sanchez-Diaz M (2008). Involvement of abscisic acid and polyamines in berry ripening of Vitis vinifera (L.) subjected to water deficit irrigation. Australian Journal Of Grape And Wine Research..

[54] Yoshida T (2010). AREB1, AREB2, and ABF3 are master transcription factors that cooperatively regulate ABRE-dependent ABA signaling involved in drought stress tolerance and require ABA for full activation. Plant Journal..

[55] Hwang K, Susila H, Nasim Z, Jung JY, Ahn JH (2019). Arabidopsis ABF3 and ABF4 Transcription Factors Act with the NF-YC Complex to Regulate SOC1 Expression and Mediate Drought-Accelerated Flowering. Molecular Plant..

[56] Zuo X (2018). Expression of genes in the potential regulatory pathways controlling alternate bearing in ‘Fuji’ (Malus domestica Borkh.) apple trees during flower induction. Plant Physiology And Biochemistry..

[57] Flores HE, Galston AW (1982). Analysis of polyamines in higher plants by high performance liquid chromatography. Plant physiology..

[58] Ma L, Zhang X, Meng Y, Zhao J, Zhang M (2018). Influence of Spraying GA(3) and 6-BA on Endogenous Hormone Content and the Flowering Rate of “Fuji” Apple. Acta Botanica Boreali-Occidentalia Sinica..

[59] Gambino G, Perrone I, Gribaudo I (2008). A Rapid and Effective Method for RNA Extraction from Different Tissues of Grapevine and Other Woody Plants. Phytochemical Analysis..

[60] Livak KJ, Schmittgen TD (2001). Analysis of relative gene expression data using real-time quantitative PCR and the 2(T)(-Delta Delta C) method. Methods..

